# The Implications of Vaccines in Older Populations

**DOI:** 10.3390/vaccines10030431

**Published:** 2022-03-11

**Authors:** Jean-Pierre Michel, Emilia Frangos

**Affiliations:** Département de Réhabilitation et Gériatrie, Hôpitaux Universitaires de Genève, 1226 Geneva, Switzerland; emilia.frangos@hcuge.ch

**Keywords:** vaccines, old, aged, cardio-vascular, dementia, antimicrobial resistance

## Abstract

Mean longevity is increasing worldwide, with major consequences for public health worldwide, as the global population of adults aged over 65 years now exceeds the number of children under 5 for the first time in history. The ageing process over the life course is extremely heterogeneous, and it will be important to promote and enhance healthy ageing worldwide. Vaccination is a key player in the healthy ageing process, both at the individual and the community level. We review here the contribution of vaccines to individual and community health. At an individual level, we highlight the prevention of infectious diseases, as well as other, less well-known benefits of vaccination, such as modulation of the inflammatory process. We then underline the importance of vaccination in achieving herd immunity and reducing the transmission of pathogens in the community. Finally, at a community level, another important benefit of vaccination is the reduction in antimicrobial resistance. Taken together, these effects contribute to ensuring the best health, for the greatest number, for the longest time possible.

## 1. Introduction

Mean longevity worldwide increased from 52.6 years in 1960 to 72.6 years in 2020 [1]. On a global scale, the number of adults aged over 65 rose from 0.17 billion in 1960 to 0.73 billion in 2020, and for the first time in history, has now exceeded the number of children under the age of 5 [2].

These demographic trends must not blur the fact that the dynamic, complex, and inescapable ageing process over the life course is extremely heterogeneous. For instance, currently, in a few African countries, life expectancy is still below 60 years (e.g., 54 years in Sierra Leone and Nigeria, 58 in South Sudan) [1], while inhabitants of the European Union (EU) can expect to live 25 years longer. Indeed, the mean life expectancy in the EU is currently 81 years. Moreover, again in the EU, the percentage of adults over 80 years of age is expected to rise from 5.2% at present to 10% in 2050 [2]. Inequities also exist between genders in the EU, with a mean time spent in disability of 14.7 years for men and 19.4 years for women [3]. 

These data strongly suggest the need to intensify consensual public health policy around the whole world to promote and enhance healthy ageing World [4]. The definition of this concept has evolved from simply “ageing free of disease, injury, and disability” to the more comprehensive definition from 2015, that defines healthy ageing as “the process of developing and maintaining functional ability to allow well-being in the very old age” [5].

In this evolving context, it is of prime importance to recall the WHO estimates that vaccines prevent more than 2.5 million deaths from communicable diseases worldwide each year. Moreover, it is crucial to note that 200 children die for vaccine-preventable diseases (VPD) each year versus 70,000 adults—an incredible 350-fold difference [6]. As stated by the renowned American vaccinologist Gregory Poland, “this imbalance is striking and reflects a number of underlying structural, economic, cultural, and political issues” [7].

In these troubled times of the COVID 19 pandemic, these various data resonate and serve as a reminder of the valuable contribution to humanity and life expectancy of life-course immunization programs [8]. The crucial contribution of vaccines to the healthy ageing process [9,10], both at the individual and community level, needs to be highlighted.

## 2. At the Individual Level, the Invaluable Anti-Infectious History of Vaccines Is Too Often Forgotten

The “still recent” long-term eradication of smallpox in 1980 and poliomyelitis in 2016 must remain foremost in our minds. These tremendous achievements were the result of exemplary public policy that made it possible to achieve high levels of population immunity in all regions around the world over a prolonged period, with adequate surveillance in place [11]. Moreover, from the pre-vaccine era to modern times, in Australia, Canada, the United Kingdom, and the US, incident mortality and morbidity of numerous other infectious diseases (such as rubella, diphtheria, Hemophilus influenzae, measles, mumps, pertussis, and tetanus) have decreased drastically, by 90% [12]. The elimination of these diseases is not complete, mainly because of too low or decreasing vaccination rates, as has been clearly illustrated for measles [13] and pertussis [14], with their recent outbreaks. Diseases that have an environmental reservoir (e.g., tetanus) and diseases that have animal reservoirs (e.g., Japanese encephalitis and rabies) could conceivably be eradicated on a global scale, but only if high uptake of vaccination is achieved and maintained in humans (and animals, in the case of rabies) [11]. Even in these conditions, and before the COVID 19 pandemic, vaccines saved more than 2.5 million lives worldwide every year by preventing communicable diseases [4], whose incidence decreased from 33% in 1990 [15] to 19% in 2016 [16]. Indeed, before the COVID pandemic, in parallel to a proven decrease in infectious disease incidence, there was certainly also a reduction in infection-related disability, as it is known that communicable diseases are the sixth most common cause of functional decline.

To summarize, a quote from Plotkin is fitting, who affirmed that “history confirms that vaccines have been the medical intervention with the greatest beneficial impact on human health and longevity” [17].

## 3. At the Community Level, the Importance of Community Immunity Is Still Poorly Understood

The COVID-19 pandemic has revealed all too clearly that the current individual way of thinking and acting raises major issues for people to be vaccinated. While many vaccines accepters value vaccination for the benefit of themselves and others, a large majority follow recommendations from their governments and agencies without giving any thought to the community-wide benefits [18]. However, the historical clues and scientific randomized controlled studies are indisputable and provide convincing evidence. The eradication of smallpox and poliomyelitis was followed by numerous positive attempts at the elimination of other infectious diseases. For example, conscious of the very high mortality rate from pneumonia and influenza in the general Japanese population, the country’s government proposed a mandatory school-based trivalent influenza vaccination program in 1962. Within two decades, the excess death rate fell from 14 per 100,000 to fewer than 3 per 100,000, attesting to the great efficacy of immunizing children to protect the general population. Unfortunately, yielding to incessant parental pressure, the program was stopped in 1987, resulting in a rapid rebound of mortality from pneumonia and influenza in the Japanese population (rising again to 10 per 100,000 by 1998) [19].

A more recent randomized cluster trial involving 947 Canadian children (>3 and <15 years) among a Hutterite community of 2326 persons living in 2 villages confirmed the importance of herd immunity. The intervention group (*n* = 502) received the influenza vaccine and was compared with the control group (*n* = 455), which received the hepatitis A vaccine. The incidence of PCR-confirmed influenza A decreased by 59% in the general community of the intervention group, testifying once against that flu vaccination of children significantly decreases the spread of the virus within the whole population [20].

The third example comes from a systematic review of the literature on the indirect (herd) protection afforded by conjugate pneumococcal vaccination (PCV13) in children [21]. Whatever the country studied, the global incidence of invasive pneumococcal diseases decreased by between 2.22% and 61.12% after the introduction of PCV13, highlighting again that adults aged over 65 seem to benefit most from the introduction of PCV vaccination in children [21].

Another important issue is related to herd immunity, which corresponds to the reduction of infection or disease in the unimmunized segment as a result of immunizing a proportion of the population [22]. In an observational study over a period of 3 months, in 40 nursing homes that were matched for size, vaccine uptake among the personnel in the previous flu season, and disability index of the residents, influenza vaccine was administered to volunteer staff in half the participating nursing homes, while there was no intervention in the remainder. After 3 months, multivariate analysis found a statistically significant 20% reduction in all-cause mortality among residents in the nursing homes where the staff had been vaccinated (odds ratio 0.80, 95% confidence interval (CI) 0.66–0.96, *p* = 0.02) [23].

These four examples highlight the usefulness of vaccines in halting the transmission of pathogens between generations and between healthcare workers and nursing home residents. Vaccination, therefore, contributes to improving health among millions of people through community protection. This effect is also visible in a cross-sectional manner between immunized and non-immunized individuals (i.e., non-vaccinated persons or non-responders to vaccination) of the same generation [24].

## 4. At the Individual Level, the Other Benefits of Vaccines Are Less Well Known, Particularly Those Modifying the Inflammatory Processes

Recent epidemiological studies have demonstrated the potential effect of rotavirus vaccination of children on the incidence of type 1 diabetes. After mandatory rotavirus vaccination was introduced in Australia [25] and in the US [26], it was noticed in 2019 that the incidence of type 1 diabetes decreased by 15 to 41% in the entire rotavirus vaccinated series of children, compared to children who did not receive the vaccine during the same time period. This important childhood finding will totally modify the healthcare needs and wellbeing of millions of (future) adults.

A potential role of the influenza vaccine in protecting against atherosclerosis was first suggested at the start of the 21st century [27]. The association was subsequently confirmed by randomized controlled studies [28,29], prompting the American Heart Association and American College of Cardiology to recommend the trivalent influenza vaccine as a component of secondary prevention in persons with coronary and other atherosclerotic vascular disease, with a class IB recommendation [30]. Since these recommendations, divergent results have been published regarding the effect of influenza vaccination on vascular disease outcomes. However, a recent meta-analysis confirmed firstly that lab-diagnosed influenza, influenza-like illness, and respiratory tract infection multiply the incidence of myocardial infarction by two, and secondly, that flu vaccination reduces the risk of myocardial infarction by 29% [31].

Two studies from the Taiwan Longitudinal Health Insurance Database (1996–2008) have recently investigated the potential of the influenza vaccine to prevent cardiovascular events. One study involved 7722 patients aged over 55 years with chronic obstructive pulmonary disease [32], and the second involved 4406 patients of the same age with chronic kidney disease (and without known cardiovascular disease) [33]. Both studies observed a significant decrease in cumulative event rates for acute coronary syndrome in vaccinated versus non-vaccinated individuals (*p* < 0.001). In addition, persons who received four influenza vaccines during the study period had a greater reduction in the odds of hospitalization for acute coronary syndrome than those who received only one influenza vaccine (*p* < 0.001) [32,33].

These findings are corroborated by the results of a nationwide cohort study from Denmark that included adults (>18 years) diagnosed with heart failure between January 2003 and June 2015 (*n* = 134,048). After the diagnosis of heart failure, a single flu vaccine sufficed to yield a significant reduction in cardiovascular mortality (HR = 0.82 [0.81–0.84]), with a greater protective effect observed among those who received four vaccines (HR = 0.72 [0.69–0.74]) (*p* < 0.001) [34].

Similarly, there is a growing body of evidence supporting an increased risk of stroke after the clinically proven onset of herpes zoster (HZ) infection in adults aged 50 to 60 years [35,36]. It has been shown that HZ vaccination has a protective effect in a nationwide US cross-sectional telephone survey of 265,568 non-institutionalized adults aged 50 to 79 years old [37]. By Cox proportional hazards analysis, the authors observed that individuals who had not received zoster vaccination were at a significantly higher risk of stroke compared to those who had received the live attenuated HZ vaccine (HR = 1.73, 95% CI: 1.71, 1.76) [37].

This cumulating evidence underscores the net benefit of flu vaccination in preventing cardiovascular events (coronary artery disease, heart failure, and cardiovascular death). Similarly, herpes zoster vaccination reduces the incidence of stroke post HZ infection. Clearly, the beneficial effects of vaccination on cardiovascular events in older populations have not received the attention they deserve [10].

Other potential benefits may also come to light in the future. The prospective Canadian Study of Health and Aging included a community sample of 3865 cognitively impaired participants (Alzheimer’s disease, AD) aged 65 years and older [38] to investigate the possible relation between the cognitive status of participants and previous vaccination, at baseline (1991–1992) and during follow up (1996–1997). After adjustment for age, the authors observed a significant relationship between exposure to diphtheria/tetanus, polio, and flu vaccines, and a decreased risk of AD [38]. These surprising findings highlighting the impact of life-course vaccination on chronic diseases in adulthood gave rise to the “infectious hypothesis” of late-onset dementia, which has been much debated in the literature [39,40,41]. Recently a multi-center retrospective cohort study including 12,185 bladder cancer patients with a follow-up of 3.5 to 7 years shows that the 2301 BCG-treated patients manifested more than 4-fold less risk for AD than those not treated with BCG [42,43]. On the other hand, the antituberculosis vaccine bacillus Calmette-Guérin (BCG) appears as preventative of the severity of coronavirus disease 2019 (COVID-19) [44,45]. From a life-course perspective, the role of infant vaccinations and boosters is fundamentally important in contributing to maintaining good health well into adulthood.

## 5. At the Community Level, the Most Promising Effect of Vaccines Would Be the Reduction of Antimicrobial Resistance

There are several measures that have been well-described to control antimicrobial resistance. One additional path towards eradicating resistance is the development of new antimicrobial agents and new vaccines [46]. Unfortunately, detection of pathogens that are resistant to antimicrobial drugs occurs earlier and earlier, whereas there is a paucity of new antibiotics [47]. Conversely, vaccines do not acquire significant resistance, thus making them an attractive solution to fight against antimicrobial resistance. This is a two-pronged approach, whereby vaccines first protect people against infections, such as influenza or pneumococcal pneumonia, and second, they help to reduce the spread of disease, and consequently, reduce the need for antibiotics. The herd immunity achieved in this way is not only beneficial for its effects on those who are vaccinated, but it also protects those who cannot be immunized, such as the immunocompromised. In parallel to the introduction of multivalent anti-pneumococcal vaccines, there has also been a decrease in the density of oral carriage of the microbes, thereby reducing genetic exchanges of resistance. Nevertheless, since nature abhors a vaccine, a few serotypes that are not contained in the vaccine (A19) have emerged. Two years after the introduction of the 13-valent pneumococcal conjugate vaccine (PCV13) in 2010, resistance against macrolides, cephalosporines, tetracyclines, and penicillin decreased respectively by 63, 81, 81, and 83% [46]. Currently available vaccines are therefore excellent tools to fight against antimicrobial resistance, although it remains necessary to increase access to vaccines, coverage rates, and the development of new vaccines. An impact study on the clinical prescription of antibiotics and reduction in antimicrobial resistance has already been included in all new vaccine trials by the WHO [48].

## 6. Conclusions

The contribution of vaccines to promoting healthy ageing needs to be valued more highly. At the individual level, life course vaccination programs contribute substantially to reducing the global burden of infectious diseases, decreasing mortality and—a less known effect—to preventing infection-related disability. Beyond this well-known but too often forgotten efficacy, it was also recently discovered that at the individual level, vaccines can reduce type 1 diabetes (rotavirus vaccine), prevent myocardial infarction and all-cause mortality in heart failure patients (influenza vaccine), and lower stroke incidence (herpes zoster vaccine). Moreover, when vaccine uptake is high enough, community immunity protects non-vaccinated and immune-compromised individuals (influenza and pneumococcal vaccines) and reduces antimicrobial resistance (pneumococcal vaccine). Despite the occurrence of rare, mild, and usually self-limiting side effects of vaccines, public health deciders need to strongly promote vaccination to favor healthy ageing.

## Data Availability

Not applicable.
